# Patterns of Physical Activity Among University Students and Their Perceptions About the Curricular Content Concerned With Health: Cross-sectional Study

**DOI:** 10.2196/31521

**Published:** 2022-04-29

**Authors:** Arun Kumar Verma, Girish Singh, Kishor Patwardhan

**Affiliations:** 1 Department of Kriya Sharir Faculty of Ayurveda, Institute of Medical Sciences Banaras Hindu University Varanasi India; 2 Centre of Biostatistics Institute of Medical Sciences Banaras Hindu University Varanasi India

**Keywords:** physical activity, university students, university, exercise, students, inactive, curricula, healthy lifestyle, higher education

## Abstract

**Background:**

University students are at risk of losing their focus on maintaining healthy levels of physical activity because of their engagements with curricular and cocurricular activities. In India, the physical activity levels of the adult population have been reported to be declining in the recent years. However, studies focusing on university students pertaining to their physical activity are lacking in the Indian context. Moreover, a question that has not been properly addressed is the following: “do the curricula in higher education promote physical activity?”

**Objective:**

Our paper aims at describing the physical activity levels of the students in a large public-funded central university located in northern India. The study also aims at capturing the student perceptions about the emphasis they receive on leading a physically active lifestyle during their routine curricular activities.

**Methods:**

This is a cross-sectional descriptive study and uses International Physical Activity Questionnaire—Long Form to record physical activity among 4586 students. Stratified sampling method was used to enroll the students from each stream (faculty). Out of 30,667 students, about 15% were included from each faculty. The study was conducted between 2016 and 2019. To capture the student perceptions, we used a newly developed 5-item scale.

**Results:**

From a total of 4586 participants in the study, 2828 (61.7%) were male and 1758 (38.3%) were female students. The mean age of our sample was 22.34 (SD 3.12) years. Our results indicate that about 14.5% (n=666) of all students in the study fall under the “Inactive” category. Furthermore, the perception about the curricular content pertaining to physical activity varied widely between the students of different streams.

**Conclusions:**

Our sample reported a better physical activity pattern in comparison to the reported overall physical activity levels of the adult population of India. Our results also suggest that health-related topics are inadequately represented in many of the streams of higher education in the university.

## Introduction

### Background

Patterns of physical activity are undergoing significant change in the recent years among individuals of all age groups across the globe [1-5]. Literature suggests that these changes are mostly influenced by factors such as changing lifestyles, gender differences, economic status, sociocultural influences, educational levels, occupational factors, and other determinants [6,7]. Many workers in the field have reported a declining trend in physical activity profile among children, young adults, and adults across different societies including India [8-11]. An increased engagement with virtual games, cell phones, television, computers, and social media are possibly some of the important contributing factors to this trend among youth. Increased use of vehicular mode of transportation and reduced involvement in outdoor activities also contribute to this outcome [12-15]. Further, the incidence of health conditions such as being overweight, obesity, coronary artery disease, hypertension, diabetes mellitus, and depression are known to have increased among young adults, and a suboptimal physical activity has been recognized to be an important factor associated with these conditions [16-23]. According to the World Health Organization (WHO), more than a quarter of the world’s adult population are insufficiently active, and around 1 in 3 women and 1 in 4 men do not do enough physical activity to stay healthy [24]. WHO recommends various levels of physical activity for people belonging to different age groups [24].

### Trends in Physical Activity

It would be pertinent to understand the trends in physical activity that have been reported in India and elsewhere. In a study conducted by the Indian Council of Medical Research, physical activity patterns in adults across India were studied. The research reported that out of the 14,227 individuals studied, 54.4% (n=7740) were inactive, 31.9% (n=4538) were active, and 13.7% (n=1949) were highly active [9]. This trend is a matter of concern as the percentage of inactive population appears to be very significant.

There are several studies to show that the decreasing physical activity levels among youth are a matter of concern in many countries. Physical activity patterns among university students have received some attention in the recent years across the globe [25-29]. A study on European university students from 13 countries investigated the trends of smoking, diet, physical exercise, and attitudes toward health. The study compared these trends between the results of 2 surveys carried out in 1990 and 2000 and suggests that differences in health behaviors, beliefs, and risk awareness were disappointing [25]. In another study, 259 medical students in the age group of 18-22 years were interviewed using the International Physical Activity Questionnaire (IPAQ) in Bangalore. The study reported that 41.3% showed high levels of physical activity, 43.2% showed moderate, and 15.4 % of students showed low level of physical activity respectively [26]. Another study was conducted among 100 students in the Health Science faculty at a private university in Lebanon. The investigators report that most of the students did not consume a healthy diet and that they don’t exercise as much as they like to [27]. A study among 334 students at the Alexandru Ioan Cuza University from Iasi, Romania conducted using the International Physical Activity Questionnaire—Long Form (IPAQ-L) reported that the lifestyle and physical activity levels were reasonably good, and the overall average metabolic equivalent (MET) minutes per week were 5343.92 (SD 2314.02) [28]. A study among 297 undergraduate students from 20 to 22 years of age from the University of Maribor reported that 79.8% of students were insufficiently physically active according to the WHO recommendations [29]. In another study, weekly physical activity scores of the students from sports departments and non–sport departments were compared among 300 university students in Turkey. The results revealed that the students from sports departments performed better than others in terms of weekly total physical activity [30].

However, there are no large and systematically performed studies available to show if the overall physical activity levels are comparable with the recommended ones among students at Indian universities. This question becomes important considering the fact that universities are the places where health awareness is supposed to be inculcated among the youth, and these students are at the risk of losing focus on physical activity because of the burden of curricular activities.

Similarly, studies exploring the curricular content and student perceptions about the motivation and information they receive during their routine curricular activities with reference to leading a physically active lifestyle are scarce. However, a few studies have shed light on the perceptions of student population toward health-related information in the curriculum. Nevertheless, these perceptions among students vary from one setting to another [31-34]. Such studies are not available in an Indian context. This question becomes important keeping in mind the diverse nature of the Indian education system and curricula.

The increasing involvement of the student population with mobile phones, computers, social media, and virtual games has had a negative impact on physically active lifestyles. This has also had an impact on mental health status among the youth. Increased use of mobile devices also has been said to be responsible for increasing sleep-related and circadian rhythm–related disorders. The addictive nature of these platforms is a worrisome aspect [35-37].

Hence, we planned this study to understand the physical activity trends among the students at Banaras Hindu University (BHU) and to capture their perceptions regarding the curricular content related to physical activity. BHU is a public central university located in Varanasi, Uttar Pradesh, established in 1916. It is one of the largest residential universities in Asia. BHU is organized into 6 institutes and 14 faculties (streams) and 144 departments. The total student enrollment at the university is around 30,000, and this number represents almost all states of India along with a few foreign countries.

Since the university is a public-funded one, the fee structure of the university is highly affordable, and all the admissions are based on successfully securing ranks through different all-India level screening tests held every year. For all graduate, postgraduate, and doctoral programs, there is a lot of competition for the same reason. Hence, it can be presumed that the distribution of the student population represents the society in terms of socioeconomic status because only meritorious students get to study in this university. As the university is positioned as a major learning center in the eastern part of India, the student population mostly represents this part of the country.

### Objectives of the Study

The primary objective of the study was to understand the proportion of students at Banaras Hindu University (BHU) who fall under different categories of physical activity (ie, physically inactive, active, and highly active). While analyzing the trends, we also considered the different programs under which these students are registered (undergraduate, postgraduate, or doctoral). The study also aimed at comparing the physical activity profiles of students from different faculties of BHU. Throughout the study, we aimed to understand the differences in the physical activity profiles with respect to age and gender. Another objective of the study was to map views and opinions of the students regarding the information and motivation they receive in their respective faculties and departments as a part of their routine curricular activities to keep themselves physically active.

## Methods

### Study Design and Sampling

This is a cross-sectional survey study wherein a stratified sampling technique was employed. Individual stream (eg, Humanities, Science, Social Sciences, Medicine, and Ayurveda) was considered as one stratum. We collected the details of the total number of students registered in each of the 16 streams from the offices of the respective deans. It was decided to include about 15% of all the students from each stream considering the time and other limitations. This meant approximately 4600 students, which was thought to be sufficient to draw meaningful conclusions. In this study, though we collected the data from 4733 students, we report the physical activity patterns of 4586 students as we had to delete certain entries during data processing.

### Tools Used in the Study

To record the physical activity profiles of the students, we used the IPAQ-L [38,39]. This tool has been developed by IPAQ group and is widely used in large surveys. This tool employs an indirect method of measuring physical activity based on the recall of one’s activities over the past 1 week. The purpose of this tool is to provide a common instrument that can be used to obtain internationally comparable data on health-related physical activity. Further, a newly developed 5-item questionnaire was used to record the opinions and views of the student population. This tool was designed to capture the perceptions of the students regarding the encouragement they receive in their respective faculties and departments to keep themselves physically active.

### Translation and Revalidation of IPAQ-L

The IPAQ-L is available in different languages (English, French, German, Greek, etc) but not in Hindi. Since Hindi is the common language of communication in this part of India, the questionnaire was translated from English to Hindi by a language expert. The questionnaire was then back-translated to English by another language expert and was verified for its accuracy by another team of experts in the department. Suitable corrections were made before the tool was finalized and administered. No item was deleted or added. Both the Hindi and English versions of the tool were used in the study to collect data based on student preference after verifying the accuracy.

### Development of a New Tool to Capture the Views and Opinions of the Student Population

Since the IPAQ-L is quite lengthy, the tool to capture student perceptions about curricular content dealing with health had to be very short. After discussing with the team of experts in the department, a short 5-item questionnaire was developed, which was administered to all participants in the study. These 5 items were retained from the original questionnaire, which had 10 items, after receiving feedback from an expert group. This too was administered both in Hindi and English per students’ preference. The statements (items) included in this questionnaire were as follows:

1. The curriculum of my course or courses addresses the topics related to “importance of day-to-day physical activity in maintaining health.”
2. My faculty or department promotes physical activity or sports activities among the students in an organized manner regularly.
3. I consider the sports facilities (playgrounds, sports equipment, and sports training) available in my faculty for the students to be adequate in general.
4. I keep monitoring my body weight regularly, and I am aware of the health consequences of being overweight and obesity.
5. I consider that general health–related aspects (such as diet, nutrition, and sports) are sufficiently addressed in my curriculum.

The options given for each of the questions were in the form of a 5-point Likert scale (1=Strongly Agree, 2=Agree, 3= Undecided, 4=Disagree, and 5=Strongly Disagree).

### Reliability of the New Tool

The 5-item scale was first administered to 100 students from the Institute of Agricultural Sciences for the purpose of validation. The Cronbach coefficient alpha for the scale was .725, which falls under the category of acceptable range [40]. Hence, the scale was considered as reliable.

### Ethics Approval

Ethical clearance was obtained by Institutional Ethics Committee (Reference 2014-15/EC/1323) before starting the study.

### Data Collection and Data Entry

Investigators collected the data regarding the total number of students registered from different faculties of BHU by writing to the deans. Since the information contained only numbers and not the list of students, it was decided that the required number of classes be randomly selected, and all students of those classes (batches) be administered with the tool. The first author of this paper visited different departments and received permission from concerned heads of the departments to collect the data in leisure hours from different classes. The specific classes were selected by computer-generated random sequence method. A written consent was obtained from each of the participants. Though we collected the hard copies of the filled-in questionnaires from the volunteers, to ensure precision and uniformity, we prepared an online form to enter the data. Finally, the data were downloaded in the form of a spreadsheet. The data were collected between 2016 and 2019. IPAQ-L and the 5-item questionnaire were filled simultaneously by all volunteers.

### Data Analysis

The data analysis to evaluate physical activity patterns was carried out according to the data processing rules of the IPAQ-L. The major steps involved in this process were data cleaning, excluding the outliers based on the maximum values allowed; this ensured receiving minimum values for the duration of the reported activity; truncation of data; calculating MET minutes per week scores for walking, moderate-intensity; and vigorous-intensity activities; as well as calculating the Total Physical Activity Scores. All these steps were followed per the guidelines of the IPAQ-L. The final step was to classify the entire sample into categorical data in terms of (1) low (inactive), (2) moderate (active), and (3) high (highly active) levels of physical activity.

The classification of physical activity into three levels is based on the following criteria [39,40]:

### Low Activity

No activity is reported, or some activity is reported but not enough to meet categories 2 or 3.

### Moderate Activity

Any of the following 3 criteria applies: (1) 3 or more days of vigorous-intensity activity of at least 20 minutes per day; (2) 5 or more days of moderate-intensity activity or walking of at least 30 minutes per day; or (3) 5 or more days of any combination of walking, moderate-intensity, or vigorous-intensity activities achieving a minimum of at least 600 MET minutes per week.

### High Activity

Any one of the following two criteria: (1) vigorous-intensity activity on at least 3 days and accumulating at least 1500 MET minutes per week; or (2) 7 or more days of any combination of walking, moderate-intensity, or vigorous-intensity activities accumulating at least 3000 MET minutes per week.

## Results

### Sample Characteristics

The total student strength of BHU was 30,667, and upon calculation, 15% of this population is 4600. We collected a sample of 4733. However, after excluding the outliers and erratic entries as per the IPAQ-L criteria, the sample that was analyzed included 4586 students.

Table 1 shows the distribution of participants as per their programs of study, gender, and age group. The total number of male and female students included in the study was 2828 (61.7%) and 1758 (38.3%), respectively. Mean age of the sample in the study was 22.34 (SD 3.12) years (male students: 22.37, SD 3.13 years; female students: 22.29, SD 3.12 years). Out of 4586 students, 3048 (66.4%) were from undergraduate programs, 1406 (30.7%) were from postgraduate programs, and 132 (2.9%) were from doctoral level programs.

**Table 1:** Distribution of volunteers as per age group, gender, and program of study.

Age group (years) and gender		Program of study				Total	
		PhD, n (%)	Postgraduate, n (%)	Undergraduate, n (%)	Based on gender, n		In each age group, n
**16-20**							1445
	Female	N/A^a^	7 (1.2)	553 (98.8)	560		
	Male	N/A	20 (2.3)	865 (97.7)	885		
**21-25**							2539
	Female	5 (0.5)	434 (44)	547 (55.5)	986		
	Male	14 (0.9)	633 (40.8)	906 (58.3)	1553		
**26-30**							503
	Female	17 (9.7)	107 (60.8)	52 (29.5)	176		
	Male	52 (15.9)	163 (49.8)	112 (34.3)	327		
**31-35**							82
	Female	11(36.7)	16 (53.3)	3 (10)	30		
	Male	21 (40.4)	22 (42.3)	9 (17.3)	52		
**36 and above**							17
	Female	4 (66.7)	2 (33.3)	0 (0)	6		
	Male	8 (72.7)	2 (18.2)	1 (9.1)	11		
**Total**							4586
	Female	37	840	1155	1758		
	Male	95	566	1893	2828		

^a^N/A: not applicable.

### Physical Activity Levels

Table 2 displays the overall distribution of subjects into low (inactive), moderate (active), and high (highly active) levels of physical activity. In our sample, we noted that about 666 students (14.5% of all students) fell under the low category of physical activity (407 [14.4%] male and 259 [14.7%] female students), whereas an almost equal proportion (ie, 651 students [14.2% of all students]: 269 [15.3%] female and 382 [13.5%] male students) fell under moderate physical activity category. Further, about 3269 students (71.3% of all: 2039 [72.1%] male and 1230 [70%] female) fell under high level of physical activity. The difference between physical activity levels for male and female students was statistically not significant (χ^2^_2_=3.237, *P*=0.2). Further, the difference was also not significant between male and female participants for any program of study. Table 2 also shows the distribution of volunteers into high, moderate, and low levels of physical activity based on their programs of study and gender. Among the 132 PhD scholars, 28 (21.2%) fall under the low category, 12 (9.1%) under moderate, and 92 (69.7%) under high category. Among all 1406 postgraduate students, 215 (15.3%) fall under the low, 199 (14.2%) under the moderate, and 992 (70.5%) fall under the high category. Among the 3048 undergraduate students, 423 (13.9%) fall under the low category, 440 (14.4%) under the moderate category, and 2185 (71.7%) fall under the high category. The difference between physical activity of students of various programs was not statistically significant (χ^2^_2_=8.282, *P*=.08).

Table 3 depicts the distribution of volunteers into high, moderate, and low categories based on age group. As the table suggests, the number of students in the “highly active” category is highest among lower age groups, and the number of students in the “inactive” category is highest among higher age groups. The difference between physical activity of students of different age groups was statistically significant (χ^2^_2_=35.387, *P*<.001). This indicates that as the age increases, the likelihood of indulging in physical activity decreases.

**Table 2:** Distribution of volunteers into high, moderate, and low levels of physical activity based on programs of study and gender.

Program of study and gender		Category					Comparison between gender and category
		Highly active (high), n (%)	Active (moderate), n (%)	Inactive (low), n (%)	Total, n		
**PhD**						χ^2^=1.760, *df*=2, *P*=.42	
	Female	23 (62.2)	5 (13.5)	9 (24.3)	37		
	Male	69 (72.6)	7 (7.4)	19 (20)	95		
	Total	92 (69.7)	12 (9.1)	28 (21.2)	132		
**Postgraduate**						χ^2^=5.404, *df*=2, *P*=.07	
	Female	380 (67.2)	88 (15.5)	98 (17.3)	566		
	Male	612(72.9)	111 (13.2)	117 (13.9)	840		
	Total	992 (70.5)	199 (14.2)	215 (15.3)	1406		
**Undergraduate**						χ^2^=1.522, *df*=2, *P*=.47	
	Female	827 (71.6)	176 (15.2)	152 (13.2)	1155		
	Male	1358 (71.8)	264 (13.9)	271 (14.3)	1893		
	Total	2185 (71.7)	440 (14.4)	423 (13.9)	3048		
**Total**						χ^2^=3.237, *df*=2, *P*=.2	
	Male	2039 (72.1)	382 (13.5)	407 (14.4)	2828		
	Female	1230 (70)	269(15.3)	259 (14.7)	1758		
Grand total		3269 (71.3)	651 (14.2)	666 (14.5)	4586		

**Table 3:** Distribution of volunteers into high, moderate, and low categories based on age group; overall comparison of physical activity levels among different age groups: χ2=35.387, df=2, *P*<.001.

Age group (years) and gender		Category					Comparison between male and female
		Highly active (high), n (%)	Active (moderate), n (%)	Inactive (low), n (%)	Total, n		
**16-20**					1445	χ^2^=.327, *df*=2, *P*=.52	
	Male	659 (74.5)	126 (14.2)	100 (11.3)			
	Female	402 (71.7)	86 (15.4)	72 (12.9)			
**21-25**					2539	χ^2^=2.988, *df*=2, *P*=.22	
	Male	1123 (72.3)	208 (13.4)	222 (14.3)			
	Female	686 (69.6)	155 (15.7)	145 (14.7)			
**26-30**					503	χ^2^=0.976, *df*=2, *P*=.61	
	Male	218 (66.7)	42 (12.8)	67 (20.5)			
	Female	124 (70.5)	18 (10.2)	34 (19.3)			
**31-35**					82	χ^2^=3.663, *df*=2, *P*=.16	
	Male	29 (55.8)	6 (11.5)	17 (32.7)			
	Female	16 (53.3)	8 (26.7)	6 (20)			
**36 and above**					17	χ^2^=6.783, *df*=2, *P*=.03	
	Male	10 (90.9)	0 (0)	1 (9.1)			
	Female	2 (33.4)	2 (33.3)	2 (33.3)			

### Total Physical Activity MET Distribution

Table 4 depicts the distribution of MET minutes per week under different categories in the form of total walking, total moderate activity, total vigorous activity, and total physical activity MET minutes per week. The mean total physical activity MET minutes per week for male students was 4678.5 (SD 3037.01), and for female students was 4321.4 (SD 2874.09). Overall mean total physical activity MET minutes per week was 4541.6 (SD 2980.35). The difference between the MET minutes per week among male and female students was statistically significant for all categories of physical activity domains reported as suggested by *P* values. It means that the total MET minutes per week were less among female students in comparison to their male counterparts in each domain.

**Table 4:** Distribution of METa minutes per week under different domains.

MET	Gender				Mann Whitney test *P* value
	Female (N=1758), mean (SD)	Male (N=2828), mean (SD)	Total (N=4586), mean (SD)		
Total walking MET	2107.2 (1334.39)	2201.2 (1380.27)	2165.2 (1363.49)	.02	
Total moderate MET	1387.47 (1198.08)	1498.81 (1258.63)	1456.13 (1236.81)	.004	
Total vigorous MET	826.76 (1665.84)	978.46 (1760.39)	920.31 (1726.15)	<.001	
Total physical activity MET	4321.4 (2874.09)	4678.5 (3037.01)	4541.6 (2980.35)	<.001	

^a^MET: metabolic equivalent.

### Faculties With Least Active Students

As Table 5 suggests, among all the faculties, the Faculty of Ayurveda had a maximum number of least active students (ie, n=33, 41.3%). The following faculties were next in the rank: Education 18 (26.5%), Law 49 (24.6%), Medicine 43 (18.6%), Performing Arts 32 (16.9%), Environmental Science 3 (16.7%), Management 10 (15.9%), Science 127 (14.4%), Arts 140 (13.5%), Social Sciences 59 (12.6%), Agriculture 37 (12.3%), Commerce 37 (12.2%), Women’s College 54 (12.3%), Visual arts 13 (10.9%), Sanskrit Studies 10 (6.7%), and Dental Sciences 1 (2.9%). The difference between physical activity levels in different streams was statistically significant as suggested by *P* values.

**Table 5:** Distribution of volunteers into high, moderate, and low categories based on their faculty affiliation (χ2=126.2, df=30, *P*<.001).

Faculty	Category			
	Highly active (high), n (%)	Active (moderate), n (%)	Inactive (low), n (%)	Total, n
Agriculture	226 (75.1)	38 (12.6)	37 (12.3)	301
Arts	739 (70.9)	163 (15.6)	140 (13.5)	1042
Ayurveda	33 (41.2)	14 (17.5)	33 (41.3)	80
Commerce	236 (77.6)	31 (10.2)	37 (12.2)	304
Dental Sciences	26 (76.5)	7 (20.6)	1 (2.9)	34
Education	38 (55.9)	12 (17.6)	18 (26.5)	68
Environmental Sciences	15 (83.3)	0 (0)	3 (16.7)	18
Law	133 (66.8)	17 (8.6)	49 (24.6)	199
Women’s College	333 (75.3)	55 (12.4)	54 (12.3)	442
Management	42 (66.6)	11 (17.5)	10 (15.9)	63
Medicine	142 (61.5)	46 (19.9)	43 (18.6)	231
Performing Arts	127 (67.2)	30 (15.9)	32 (16.9)	189
Sanskrit Studies	120 (81.1)	18 (12.2)	10 (6.7)	148
Science	626 (71.1)	128 (14.5)	127 (14.4)	881
Social Sciences	347 (74.3)	61 (13.1)	59 (12.6)	467
Visual Arts	86 (72.3)	20 (16.8)	13 (10.9)	119
Total	3269 (71.3)	651 (14.2)	666 (14.5)	4586

### Domains of Physical Activity Reported

Our sample reported activities for transportation using bicycle (n=2255, 49.18%), walking (n=4190, 91.37%), vigorous housework outside home (n=1196 26.10%), moderate housework outside home (n=2635, 57.46%), moderate housework inside home (n=3208, 69.97%), vigorous leisure physical activity (n=1879, 40.97%), moderate leisure physical activity (n=1993, 43.46%), and leisure time walking (n=3456, 75.36%).

### Views and Opinions of the Students

Multimedia Appendix 1 shows the responses of the students to each option to the 5-item questionnaire based on gender. The statistically significant difference was observed in the responses for item numbers 1, 3, and 4 among male and female students, whereas no statistically significant response was found for item numbers 2 and 5. Multimedia Appendix 1 shows the number and percentage responses of the students to each option to the 5-item questionnaire based on gender.

Multimedia Appendix 2 shows the responses of students to 5-item questionnaire based on the programs in which they are registered. A statistically significant difference between the responses based on the courses registered (undergraduate, postgraduate, or PhD) is observed for all 5 items.

Multimedia Appendix 3 shows the mean scores for each item in each faculty. A mean score of less than 3 for any item was considered to be indicating a positive perception about the curricular activities leading to an encouraging environment that fosters a physically active and healthy lifestyle. A mean score of more than 3 for any item was considered as indicating dissatisfaction toward the curriculum of the faculty with respect to leading a physically active lifestyle. From Multimedia Appendix 3, it becomes clear that the faculties of Agriculture Sciences, Arts, Ayurveda, Dental Sciences, Medicine, Performing Arts, and Science were those where the mean scores for any of the questions did not exceed 3 or more. Hence, it can be presumed that the students in these faculties receive some kind of motivation that leads to a physically active lifestyle as a part of their curricular activities.

## Discussion

### Physical Activity Profiles Compared With Other Studies

This is one of the first studies from India that looks at physical activity levels in a focused way among a large number of university students. According to the Indian Council of Medical Research study (2014), the total percentage of inactive adults was 54.4% in India [9]. The percentage of highly active adults was 13.7%. However, the mean age group of this study sample was around 40 years. Since our study sample belongs to a mean age of around 22 years, a true comparison of the results is not possible. However, our results are much more encouraging than the ones reported in this study. It appears from our results that younger adults are more likely to indulge in physical activity than the older people. Since our sample had a mean age of 22 years, it is likely that our sample is more physically active.

A study based on the pooled data from 358 population-based surveys from across 168 countries, including 1.9 million participants reported that the global age-standardized prevalence of insufficient physical activity was 27.5% in 2016, with a difference between sexes of more than 8 percentage points [2]. In comparison to this, our sample gives a better picture. We report only about 14.5% of inactive student population. However, our study sample is smaller, younger, and more homogenous than such studies with bigger data. Hence, the results of our study are to be viewed in this context.

Another study conducted among university students in Romania included a total of 333 students, with an age average of 21.05 (SD 1.98) years [28]. According to the results of this study, mean total physical activity MET minutes per week are almost comparable with those of our study, especially among female students. However, the average total physical activity MET minutes per week among males was better in their study than in ours. This again confirms the idea that the younger age group of adult population is more inclined toward indulging in physical activity.

Another study determined the physical activity performed by undergraduate students from 20 to 22 years of age, including its frequency and intensity [29]. The sample consisted of 297 students from the University of Maribor. Their results indicate that 79.8% of students were inactive; hence, our situation in BHU appears to be much better where 71% of students are highly active. These differences need further evaluation keeping the contextual differences in view.

In yet another study, the investigators investigated the physical activity and quality of life of sports department students and other department students attending university [30]. A total of 300 university students participated in this study. In comparison with the genders, the total average physical activity score of men was found to be 4938.86 (SD 3919.33) MET minutes per week, while that of women was found to be 2592.44 (SD 2276.82) MET minutes per week. In comparison to these results, female students in our study appear to be much more physically active.

According to the results of a study consisting of 200 study subjects, 59% were having a sedentary lifestyle, 27% were moderately active, and 14% had vigorously active lifestyle. The study was conducted among the patients attending health training centers in Nagpur, and participants’ age ranged from 40 to more than 70 years [5]. This study reported a significantly increasing trend for sedentary lifestyle with age, a finding that is consistent with our results as well, although the age range of the subjects in our study was different. This further confirms the age-related differences in the physical activity levels.

A study conducted in urban and rural Vellore city, Tamil Nadu, assessed the prevalence and factors associated with insufficient physical activity among adults aged 30-64 years [11]. The prevalence of insufficient physical activity was in 63.3% in the urban area and 40.6% in the rural area. Though our results cannot be meaningfully compared with this study (as the sample characters are different), we report a better physical activity profile. The differences are likely to be because of differences in the mean age of the samples studied.

### Student Perceptions

Our study suggests that the student perceptions vary significantly from one stream of study to another indicating that the curricular activities of all streams do not encourage physically active lifestyle equally. The curricular activities of Agriculture Sciences, Arts, Ayurveda, Dental Sciences, Medicine, Performing Arts, and Science appear to be encouraging physical activity in one form or the other. This heterogenous perception indicates that there is a need for having a relook at all curricula to see if sufficient emphasis is placed in health-related aspects.

The growing health care burden of India is mainly due to the increasing prevalence of lifestyle-related diseases such as hypertension, obesity, diabetes, depression, and metabolic syndrome. Increasing use of sugars, fats, and other high-calorie fast foods among youth is compounding the situation. Most of these diseases are preventable if right intervention in terms of dietary pattern and regular physical activities are incorporated at the right age. An increasing use of smartphones, as well as increasing indulgence in virtual games and social media are said to be causing multiple sleep-related and cognition-related disorders [12-23].

There have been several studies where the student perceptions about various aspects pertaining to their physical activities have been evaluated. Different approaches of inculcating the habit of leading a physically active lifestyle among the student community have also been suggested [41-50]. However, the situation in India is complex owing to the presence of a variety of regulations and norms of developing curricula in higher education institutions. Similarly, there are different types of universities including deemed universities, private universities, state universities, and central universities [51]. The education policies thus far have mostly emphasized the importance of physical education in schools.

Our study suggests that various curricula of higher education have several lapses when it comes to health-related topics. Universities need to take up the initiative in making the students aware of the correct ways of leading a healthy lifestyle. Irrespective of the stream of education, keeping oneself physically and psychologically fit is essential to leading a healthy life. Our results seem to suggest that health education must become a part of all streams of higher education irrespective of the stream.

### Limitations and Other Aspects to Consider While Interpreting Our Results

Limitations and some other aspects pertaining to our findings will be enumerated in this section.

1. Since this study employs an indirect method of recording the physical activity levels based on 7-day recall, there are chances that respondents might tend to overestimate their physical activity levels.
2. The data were collected from 2016 to 2019, and the seasonal changes in the activity might have been gone unnoticed.
3. Indian Universities follow 6-day weeks, Sundays being the only holidays. This might be a reason for the higher level of weekly physical activity in the context of our study population.
4. Since BHU is a large residential university in terms of the area of the campus (4 square kilometers), a large proportion of the students reside in hostels and do not use vehicular mode of transportation for daily commute within the university. This might be another reason for a higher level of physical activity reported in general.
5. Many playgrounds are located within the campus of this university, and in the early morning and evening hours, one can see a good number of students using these playgrounds for playing sports and games. The engagement with games could be another reason why a higher level of physical activity is reported in this study. This could also be attributed to collective motivation in engagement of sports and games.
6. BHU is located in a state that is not in the forefront when it comes to economic development in comparison to many other states. WHO has observed that physical inactivity goes on increasing as the regions or countries develop economically [24]. This could be another reason for our sample having shown a relatively higher level of physical activity.

### Conclusion

In our sample, we report that about 14.5% of all students fall under the “inactive” category (14.4% among all male and 14.7% among all female students), about 71.3% of all students (72.1% among all male and 70% among all female students) fall under the “highly active” category, and about 14.2% of all students (13.5% of all male and 15.3% of female students) fall under the “active” category. In our study, we found that physical activity levels go on decreasing as the age increases (ie, students with the lowest physical activity rates belong to higher age groups, and highly active students belong to lower age groups). Our study also suggests that physical education and other aspects of health are inadequately and heterogeneously represented in university curricula. These topics are required to be incorporated into regular curricula in all streams of higher education in Indian universities.

## Appendix

Multimedia Appendix 1 Number and percentage responses of the students to each option to the 5-item questionnaire based on gender.

Multimedia Appendix 2 The number and percentage responses of students to 5-item questionnaire based on the programs in which they are registered .

Multimedia Appendix 3 Mean scores for each of the 5 items in the 5-item questionnaire for different faculties.

## Abbreviations

BHU: Banaras Hindu University
IPAQ: International Physical Activity Questionnaire
IPAQ-L: International Physical Activity Questionnaire—Long Form
MET: metabolic equivalent
WHO: World Health Organization
