# Supplementary material for: Patterns of Physical Activity Among University Students and Their Perceptions About the Curricular Content Concerned With Health: Cross-sectional Study
Source: JMIRx Med. 2022 Apr 29;3(2):e31521. doi: 10.2196/31521 (PMC10414421; doi:10.2196/31521)
Supplement: Multimedia Appendix 1 [file xmed_v3i2e31521_app1.docx]

**Multimedia Appendix 1.** Number and percentage responses of the students to each option to the 5-item questionnaire based on gender.

| Gender | Strongly agree, n (%) | Agree, n (%) | Undecided, n (%) | Disagree, n (%) | Strongly disagree, n (%) | Chi-square test and *P* value |
| --- | --- | --- | --- | --- | --- | --- |
| 1. The curriculum of my course or courses addresses the topics related to “importance of day-to-day physical activity in maintaining health.” | | | | | | |
| Female (n=1758) | 451 (25.7) | 373 (21.2) | 146 (8.3) | 503 (28.6) | 285 (16.2) | χ^2^=32.68, *df*=4, *P*<.001 |
| Male (n=2828) | 914 (32.4) | 626 (22.1) | 205 (7.2) | 648 (22.9) | 1. (15.4) |  |
| 1. My faculty or department promotes physical activity or sports activities among the students in an organized manner regularly. | | | | | | |
| Female (n=1758) | 291 (16.5) | 780 (44.4) | 204 (11.6) | 314 (17.9) | 169 (9.6) | χ^2^=3.55, *df*=4, *P*=.47 |
| Male (n=2828) | 475 (16.8) | 1201(42.5) | 329(11.6) | 506 (17.9) | 1. (11.2) |  |
| 1. I consider the sports facilities (playgrounds, sports equipment, and sports training) available in my faculty for the students to be adequate in general. | | | | | | |
| Female (n=1758) | 392 (22.3) | 606 (34.5) | 272 (15.5) | 326 (18.5) | 162 (9.2) | χ^2^=27.36, *df*=4, *P*<.001 |
| Male (n=2828) | 808 (28.6) | 879 (31.1) | 383 (13.5) | 464 (16.4) | 1. (10.4) |  |
| 1. I keep monitoring my body weight regularly, and I am aware of the health consequences of being overweight and obesity. | | | | | | |
| Female (n=1758) | 549 (31.1) | 792 (45.1) | 128 (7.3) | 170 (9.7) | 119 (6.8) | χ^2^=16.53, *df*=4, *P*=.002 |
| Male (n=2828) | 997 (35.4) | 1189(42) | 236 (8.3) | 270 (9.5) | 1. (4.8) |  |
| 1. I consider that general health-related aspects (such as diet, nutrition, and sports) are sufficiently addressed in my curriculum. | | | | | | |
| Female (n=1758) | 420 (23.8) | 501 (28.5) | 225 (12.8) | 386 (22) | 226 (12.9) | χ^2^=6.26, *df*=4, *P*=.12 |
| Male (n=2828) | 749 (26.5) | 796 (28.1) | 319 (11.3) | 573 (20.3) | 391 (13.8) |  |
