# Supplementary material for: Patterns of Physical Activity Among University Students and Their Perceptions About the Curricular Content Concerned With Health: Cross-sectional Study
Source: JMIRx Med. 2022 Apr 29;3(2):e31521. doi: 10.2196/31521 (PMC10414421; doi:10.2196/31521)
Supplement: Multimedia Appendix 2 [file xmed_v3i2e31521_app2.docx]

**Multimedia Appendix 2.** The number and percentage responses of students to 5-item questionnaire based on the programs in which they are registered.

| Program | Strongly agree, n (%) | Agree, n (%) | Undecided, n (%) | Disagree, n (%) | Strongly disagree, n (%) | Chi-square test and *P* value |
| --- | --- | --- | --- | --- | --- | --- |
| 1. The curriculum of my course or courses addresses the topics related to “importance of day-to-day physical activity in maintaining health.” | | | | | | |
| PhD (n=132) | 30 (22.7) | 38 (28.8) | 13 (9.9) | 42 (31.8) | 9 (6.8) | χ^2^=72.44, *df*=8, *P*<.001 |
| Postgraduate (n=1406) | 372 (26.5) | 248 (17.6) | 98 (7.0) | 419 (29.8) | 269 (19.1) |  |
| Undergraduate (n=3048) | 963 (31.6) | 713 (23.4) | 240 (7.9) | 690 (22.6) | 442 (14.5) |  |
| 1. My faculty or department promotes physical activity or sports activities among the students in an organized manner regularly. | | | | | | |
| PhD (n=132) | 19 (14.4) | 55 (41.7) | 23 (17.4) | 26 (19.7) | 9 (6.8) | χ^2^=104.2,  *df*=8, *P*<.001 |
| Postgraduate (n=1406) | 172 (12.3) | 537 (38.2) | 172 (12.2) | 314 (22.3) | 211 (15) |  |
| Undergraduate (n=3048) | 575 (18.9) | 1389 (45.6) | 338 (11.1) | 480 (15.7) | 266 (8.7) |  |
| 1. I consider the sports facilities (playgrounds, sports equipment, and sports training) available in my faculty for the students are adequate in general. | | | | | | |
| PhD (n=132) | 24 (18.2) | 51 (38.6) | 29 (22) | 21 (15.9) | 7 (5.3) | χ^2^=54.73,  *df*=8, *P*<.001 |
| Postgraduate (n=1406) | 326 (23.2) | 422 (30) | 187 (13.3) | 303 (21.6) | 168 (11.9) |  |
| Undergraduate (n=3048) | 850 (27.9) | 1012 (33.2) | 439 (14.4) | 466 (15.3) | 281 (9.2) |  |
| 1. I keep monitoring my body weight regularly, and I am aware of the health consequences of being overweight and obesity. | | | | | | |
| PhD (n=132) | 52 (39.4) | 58 (43.9) | 9 (6.8) | 10 (7.6) | 3 (2.3) | χ^2^=35.60,  *df*=8, *P*<.001 |
| Postgraduate (n=1406) | 425 (30.3) | 612 (43.5) | 105 (7.5) | 151 (10.7) | 113 (8) |  |
| Undergraduate (n=3048) | 1069 (35.1) | 1311 (43) | 250 (8.2) | 279 (9.2) | 139 (4.5) |  |
| 1. I consider that general health-related aspects (such as diet, nutrition, and sports) are sufficiently addressed in my curriculum. | | | | | | |
| PhD (n=132) | 28 (21.2) | 38 (28.8) | 21 (15.9) | 30 (22.7) | 15 (11.4) | χ^2^=44.38,  *df*=8, *P*<.001 |
| Postgraduate (n=1406) | 364 (25.8) | 336 (23.9) | 140 (10) | 336 (23.9) | 230 (16.4) |  |
| Undergraduate (n=3048) | 777 (25.4) | 923 (30.3) | 383 (12.6) | 593 (19.5) | 372 (12.2) |  |
