# Supplementary material for: Patterns of Physical Activity Among University Students and Their Perceptions About the Curricular Content Concerned With Health: Cross-sectional Study
Source: JMIRx Med. 2022 Apr 29;3(2):e31521. doi: 10.2196/31521 (PMC10414421; doi:10.2196/31521)
Supplement: Multimedia Appendix 3 [file xmed_v3i2e31521_app3.docx]

**Multimedia appendix 3.** Mean scores for each of the 5 items in the 5-item questionnaire for different faculties.

| Faculty | Number of students, n | Q1, mean (SD) | Q2, mean (SD) | Q3, mean (SD) | Q4, mean (SD) | Q5, mean (SD) |
| --- | --- | --- | --- | --- | --- | --- |
|  |  | The curriculum of my course or courses addresses the topics related to “importance of day-to-day physical activity in maintaining health.” | My faculty or department promotes physical activity or sports activities among the students in an organized manner regularly. | I consider the sports facilities (playgrounds, sports equipment, and sports training) available in my faculty for the students to be adequate in general. | I keep monitoring my body weight regularly, and I am aware of the health consequences of being overweight and obesity. | I consider that general health-related aspects (such as diet, nutrition, and sports) are sufficiently addressed in my curriculum. |
| Agriculture | 301 | 2.61 (1.446) | 2.29 (1.222) | 2.40 (1.220) | 1.88 (.981) | 2.55 (1.314) |
| Arts | 1042 | 2.66 (1.569) | 2.71 (1.335) | 2.43 (1.360) | 2.15 (1.177) | 2.71(1.497) |
| Ayurveda | 80 | 2.21 (1.144) | 2.75 (0.921) | 2.51 (1.055) | 2.08 (0.823) | 1.96 (0.878) |
| Commerce | 304 | 3.45 (1.425) | 2.82 (1.316) | 3.07 (1.489) | 1.90 (1.055) | 3.09 (1.417) |
| Dental Sciences | 34 | 2.32 (1.224) | 2.68 (1.121) | 2.68 (1.224) | 1.91 (0.866) | 2.29 (1.142) |
| Education | 68 | 2.93 (1.308) | 2.79 (1.140) | 2.94 (1.170) | 2.09 (1.168) | 3.43 (1.262) |
| Environmental Sciences | 18 | 3.28 (1.364) | 3.22 (1.215) | 3.17 (1.150) | 1.89 (1.132) | 2.72 (1.364) |
| Law | 199 | 3.20 (1.337) | 3.34 (1.203) | 3.31 (1.223) | 2.37 (1.143) | 3.22 (1.238) |
| Women’s College | 442 | 3.02 (1.363) | 2.27 (1.096) | 2.30 (1.118) | 2.07 (1.135) | 2.58 (1.276) |
| Management | 63 | 3.56 (1.254) | 2.21 (0.970) | 2.63 (1.140) | 1.95 (0.974) | 2.81 (1.203) |
| Medicine | 231 | 2.19 (1.249) | 2.49 (1.219) | 2.73 (1.361) | 2.03 (1.067) | 2.26 (1.266) |
| Performing Arts | 189 | 1.31 (0.821) | 2.15 (0.686) | 2.26 (1.234) | 2.11 (0.978) | 2.08(1.471) |
| Sanskrit Studies | 148 | 2.58 (1.498) | 2.60 (1.165) | 2.45 (1.039) | 2.30 (1.292) | 2.70 (1.532) |
| Science | 881 | 2.81 (1.439) | 2.48 (1.180) | 2.25 (1.180) | 1.90 (0.964) | 2.51 (1.240) |
| Social Sciences | 467 | 2.93 (1.559) | 3.04 (1.300) | 2.67 (1.394) | 2.57 (1.390) | 3.11 (1.477) |
| Visual Arts | 119 | 2.71 (1.485) | 2.74 (1.374) | 2.96 (1.440) | 2.08 (1.222) | 2.77 (1.362) |
| Total | 4586 | 2.75 (1.492) | 2.62 (1.250) | 2.52 (1.309) | 2.10 (1.136) | 2.69 (1.397) |
